# Efficacy and safety of reduced‐dose chemotherapy plus immunotherapy in patients with lung squamous cell carcinoma: A real‐world observational study

**DOI:** 10.1002/cam4.6478

**Published:** 2023-09-07

**Authors:** Ganlu Ouyang, Yanyang Liu, Jiewei Liu, Lin Huang, Feng Luo, Lu Li

**Affiliations:** ^1^ Department of Medical Oncology, Cancer Center, West China Hospital Sichuan University Chengdu Sichuan China; ^2^ Lung Cancer Center, West China Hospital Sichuan University Chengdu Sichuan China

**Keywords:** chemotherapy, immunotherapy, lung squamous cell carcinoma, reduced dose, toxicity

## Abstract

**Background:**

Recently, chemotherapy plus immunotherapy has achieved remarkable efficacy in lung squamous cell carcinoma (LUSC). However, some patients, especially frail people, cannot tolerate full‐dose chemotherapy in the real world. To reduce toxicity, appropriate dose reduction in chemotherapy is necessary. Therefore, this study aimed to demonstrate the efficacy and safety of reduced‐dose chemotherapy plus immunotherapy in LUSC patients in the real world.

**Methods:**

A real‐world observational study was conducted concerning patients who received chemotherapy plus immunotherapy in our situation. The primary endpoints were objective response rate (ORR) and disease control rate (DCR), and the secondary endpoints were progression‐free survival (PFS), overall survival (OS), and toxicity.

**Results:**

Between December 2018 and January 2022, 110 patients were enrolled, of whom 54 patients were chemotherapy reduced‐dose group and 56 patients were chemotherapy standard‐dose group. The ORR in the reduced‐dose group is similar to standard‐dose group (85.19% vs. 71.43%, *p* = 0.082). Similar DCR were observed (100% vs. 94.64%, *p* = 0.086). Median PFS was 12 months in the reduced‐dose group and standard‐dose group, respectively. Median OS was 15 months and 16 months in the reduced‐dose group and standard‐dose group, respectively. We reported a lower incidence of grade 3–4 toxicity in the reduced‐dose group compared with standard‐dose group (27.78% vs. 42.86%, *p* = 0.100). The major toxic reactions were better alleviated in the reduced‐dose group than in the standard‐dose group, especially in the thrombocytopenia (*p* = 0.044), peripheral nerve damage (*p* = 0.001), gastrointestinal reactions (*p* < 0.0001), and fatigue (*p* = 0.001).

**Conclusions:**

The modified regimen with attenuated doses of chemotherapy in combination with immunotherapy was effective and well tolerated in patients with LUSC. The efficacy of this modified regimen is similar to that of the full‐dose regimen.

## 1 INTRODUCTION

Lung squamous cell carcinoma (LUSC) constitutes approximately 25%–30% of non‐small cell lung cancers (NSCLCs)^1^, ^2^, ^3^ and exhibits a worse prognosis than lung adenocarcinoma (LUAD). Consequently, disease management is more challenging.^3^ Owing to their unique molecular characteristics, few druggable targets have been identified for LUSC.^4^, ^5^ Recently, investigators have explored the inhibition of programmed cell death receptor 1 (PD‐1) and its ligand (PD‐L1) in NSCLC.^6^, ^7^, ^8^, ^9^ Several randomized controlled trials have demonstrated that combining standard platinum‐based chemotherapy regimens with PD‐1/PD‐L1 inhibitors yields significant survival benefits in patients with advanced LUSC. Compared with PD‐1/PD‐L1 inhibitors alone, chemotherapy plus immunotherapy could result in a higher objective response rate (ORR) and faster tumor reduction because the efficacy of this combination is not completely dependent on PD‐L1 expression.^8^, ^10^, ^11^, ^12^, ^13^ Based on these results, the National Comprehensive Cancer Network (NCCN) and the Chinese Society of Clinical Oncology (CSCO) recommend platinum‐based chemotherapy plus immunotherapy as the standard first‐line regimen for advanced LUSC.^14^

However, chemotherapy and immunotherapy have certain limitations, particularly for some patient groups, such as elderly and frail patients, who may be unable to tolerate full‐dose chemotherapy and immunotherapy owing to serious toxicity and individual differences in clinical practice. Combining chemotherapy with immunotherapy can result in increased toxicities from both treatments. Elderly patients and those with a poor performance status (PS)^15^, ^16^ may struggle to tolerate full‐dose chemotherapy, especially when combined with PD‐1/PD‐L1 inhibitors, because they are more likely to have complications, frailty, and weaker bone marrow function. Consequently, this patient population continuously remains understudied.^17^ Therefore, individualized and precise treatments should be considered for these populations.

In the KEYNOTE‐407, RATIONALE‐307, CameL‐SQ, and ORIENT‐12 studies, patients received the following chemotherapy regimens: carboplatin (area under the concentration [AUC] of 6, Day 1) plus paclitaxel (200 mg/m^2^, Day 1) or nab‐paclitaxel ((100 mg/m^2^, Days 1, 8, and 15), q3W, 4 cycles; carboplatin (AUC of 5, Day 1) plus paclitaxel (175 mg/m^2^, Day 1)) or nab‐paclitaxel (100 mg/m^2^, Days 1, 8, and 15), q3W, 4–6 cycles; carboplatin (AUC of 5, Day 1) plus paclitaxel (175 mg/m^2^, Day 1), q3W, 4–6 cycles; gemcitabine (1.0 g/m^2^, Days 1 and 8) plus cisplatin (75 mg/m^2^, Day 1) or carboplatin (AUC of 5, Day 1), q3W, 4–6 cycles. Despite these regimens, toxicities grade ≥3 were reported in 55.7%–86.6% of patients in these RCTs.^10^, ^11^, ^12^, ^13^, ^18^ Physicians usually administer reduced‐dose chemotherapy or reduce the number of chemotherapy cycles to mitigate toxicity. However, the benefits of reduced‐dose chemotherapy combined with immunotherapy for patients with LUSC remain unreported, requiring further exploration.

Recent literature indicates that reduced‐dose chemotherapy is as effective as full‐dose chemotherapy in elderly patients.^17^, ^19^ In addition, some studies have demonstrated that reduced‐dose chemotherapy can enhance antigen release, remodel the active LUSC immune microenvironment, and increase synergistic effects.^20^, ^21^, ^22^ Consequently, we hypothesized that combining reduced‐dose chemotherapy with immunotherapy might benefit patients with LUSC while reducing toxicities. However, the use of reduced‐dose chemotherapy with PD‐1/PD‐L1 inhibitors lacks definitive evidence to support its efficacy.

Therefore, we conducted a real‐world observational study on combination therapy with reduced‐dose chemotherapy and immunotherapy in patients with LUSC. This study aimed to evaluate the efficacy and toxicity of the modified treatment regimen.

## 2 MATERIALS AND METHODS

The Ethics Committee on Biomedical Research of the West China Hospital of Sichuan University approved this study, and it was conducted according to the principles of the Declaration of Helsinki. Written informed consent was obtained from all patients.

### 2.1 Patients

Patients with LUSC who underwent chemotherapy with PD‐1/PD‐L1 inhibitors at our center were enrolled between December 2018 and January 2022. The inclusion criteria were as follows: (1) pathologically confirmed LUSC; (2) stage IV and unresectable locally advanced LUSC (stages IIIA, IIIB, and IIIC) at initial diagnosis; (3) treated with reduced‐dose chemotherapy (either reduced dose or cycles) or standard‐dose chemotherapy combined with full‐dose PD‐1/PD‐L1 inhibitors; and (4) had at least one measurable lesion on thoracic computed tomography (CT). The exclusion criteria were as follows: (1) failed follow‐up, (2) underwent chemotherapy or immunotherapy alone, and (3) did not undergo CT in our hospital and were unable to evaluate the response.

### 2.2 Treatment

The regimen for locally advanced LUSC consists of platinum‐based doublet chemotherapy combined with concurrent immunotherapy, followed by local treatment. For advanced LUSC, the first‐line regimen comprises a platinum‐based chemotherapy doublet combined with concurrent immunotherapy, followed by maintenance immunotherapy; the subsequent‐line regimen involves monotherapy chemotherapy and concurrent immunotherapy.

Chemotherapy regimens were administered at the treating physician's discretion, according to the prescription habits of our institution. The chemotherapy regimen consisted of doublet therapy with TC (paclitaxel or nab‐paclitaxel plus carboplatin); (paclitaxel or nab‐paclitaxel plus cisplatin) and GP (gemcitabine plus cisplatin), gemcitabine, or docetaxel monotherapy. The doses of PD‐1/PD‐L1 inhibitors included pembrolizumab (200 mg q3W), tislelizumab (200 mg q3W), sintilimab (200 mg q3W), camrelizumab (200 mg q3W), nivolumab (240 mg q2W or 360 mg q3W), and durvalumab (1500 mg q3W).

In the standard‐dose group, patients received the following chemotherapy doses: carboplatin (AUC of 5, Day 1) or cisplatin (75 mg/m^2^ on Days 1 to 3) plus paclitaxel (175 mg/m^2^, Day 1) or nab‐paclitaxel (260 mg/m^2^, Day 1), q3W, 4–6 cycles; gemcitabine (1000 mg/m^2^, Days 1 and 8) plus cisplatin (75 mg/m^2^ on Days 1 to 3), q3W, 4–6 cycles; gemcitabine (1000 mg/m^2^, Days 1 and 8), q3W, 4–6 cycles; and docetaxel (75 mg/m^2^, Day 1). Four‐ to six‐cycle regimens were performed, followed by local treatment or maintenance therapy with PD‐1/PD‐L1 inhibitors until disease progression, death, or unacceptable toxicity was confirmed.

In the reduced‐dose group, the patients received reduced‐dose chemotherapy and full‐dose PD‐1/PD‐L1 inhibitors. The chemotherapy dose was reduced by approximately 20% based on the standard dose, according to the actual condition of the patients. Four to six cycles or fewer than four cycles were administered, followed by local treatment or maintenance therapy with PD‐1/PD‐L1 inhibitors until disease progression, death, or unacceptable toxicity was confirmed.

### 2.3 Endpoints and toxicity evaluation

The primary endpoints were ORR and disease control rate (DCR). Tumor response was evaluated according to the Response Evaluation Criteria in Solid Tumors (RECIST 1.1) guidelines,^23^ which categorize responses as complete response (CR), partial response (PR), stable disease (SD), and progressive disease (PD). ORR was defined as CR or PR, while DCR encompassed CR, PR, and SD. Secondary endpoints included progression‐free survival (PFS), overall survival (OS), and adverse events (AEs). PFS was defined as the time from initial treatment to documented disease progression, death from any cause, or the last follow‐up visit. The OS was calculated as the time from initial treatment to death from any cause, or the last follow‐up visit. Furthermore, AE were graded according to the National Cancer Institute Common Terminology Criteria for Adverse Events (NCI‐CTCAE), version 5. Tumor imaging was scheduled at baseline, every 8 weeks during treatment, and every 12 weeks after that. Patients were contacted for survival evaluation every 12 weeks during the follow‐up period.

### 2.4 Statistical analysis

Continuous variables were summarized as medians with standard deviations, and categorical variables were described as frequencies and percentages. PFS and OS were analyzed using the Kaplan–Meier method. The hazard ratios for comparisons between the reduced‐and standard‐dose groups were calculated using a stratified Cox proportional hazards model. Subgroups were compared using the log‐rank test. Statistical significance was defined as *p* < 0.05 using a two‐tailed test. All statistical analyses were performed using R version 4.2.1.

## 3 RESULTS

### 3.1 Patient characteristics

**FIGURE 1:**
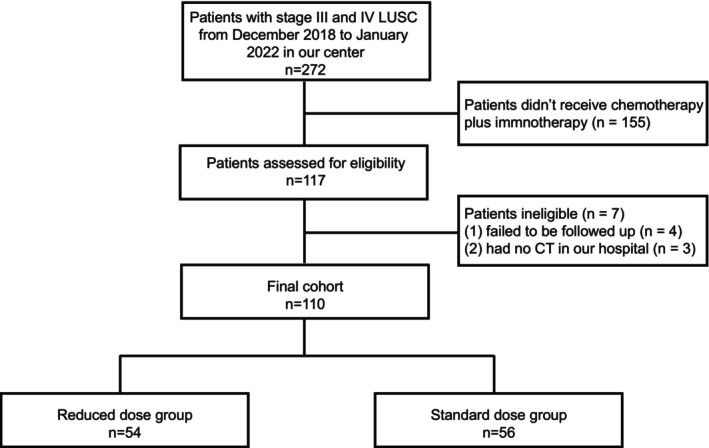
Study flow chart. LUSC, lung squamous cell carcinoma.

Among 272 patients diagnosed with stage III and IV LUSC between December 2018 and January 2022, 110 were eligible for study inclusion (Figure 1). Among these patients, 54 (49.09%) were in the reduced‐dose group, and 56 (50.91%) were in the standard‐dose group. The patient characteristics are summarized in Table 1. Patients in the reduced‐dose group were older (*p* = 0.001) and had higher Eastern Cooperative Oncology Group (ECOG) scores (*p* = 0.025) than patients in the standard‐dose group. Other baseline characteristics were similar between the two treatment groups. In both cohorts, most patients were male, smokers, and had undergone first‐line therapy. There were 49 (90.74%) and 47 (83.93%) patients who underwent double‐dose platinum‐based chemotherapy in the reduced‐dose and standard‐dose groups, respectively. Treatment regimens are listed in Table 1. In addition, most patients received 4 (range 2–6) cycles, while only one received 27 cycles of docetaxel plus nivolumab (second‐line therapy) in the standard group. In the reduced‐dose group, dose reductions were implemented in 36 (66.67%) patients before treatment was started, owing to advanced age and poor PS scores. Additionally, 11 (20.37%) underwent dose reduction after 1–2 treatment cycles because of their inability to tolerate the toxicity. Seven patients (12.96%) underwent treatment cycles reduction. Furthermore, 18 (33.33%) patients in the reduced‐dose group and 22 (39.29%) in the standard‐dose group underwent local treatment. R0 resection was performed on these patients.

**TABLE 1:** Patient characteristics.

Characteristics	Reduced‐dose group (*n* = 54)	Standard‐dose group (*n* = 56)	*p*‐value
Age, years	65 (48–81)	60 (44–78)	0.025*
Gender, *n* (%)			0.687
Male	49 (90.74%)	52 (92.86%)	
Female	5 (9.26%)	4 (7.14%)	
ECOG, *n* (%)			0.001*
0	12 (22.22%)	27 (48.21%)	
1	34 (62.96%)	29 (51.79%)	
2	8 (14.81%)	0	
BMI, %	22.21% (16.23%–29.30%)	22.18% (16.41%–27.51%)	0.802
Stage, *n* (%)			0.577
IIIA	7 (12.96%)	10 (17.86%)	
IIIB	15 (27.78%)	12 (21.43%)	
IIIC	4 (7.41%)	9 (16.07%)	
IV	28 (51.85%)	25 (44.64%)	
Smoking history, *n* (%)			0.741
Yes	42 (77.78%)	45 (80.36%)	
No	12 (22.22%)	11 (19,64%)	
Therapy line, *n* (%)			0.373
First	49 (90.74%)	48 (85.71%)	
Second	5 (9.26%)	5 (8.93%)	
Third	0	3 (5.36%)	
Chemoimmunotherapy regimen, *n* (%)			0.072
TC/TP + ICI	48 (57.14%)	42 (75.00%)	
GP + ICI	1 (1.85%)	5 (8.93%)	
Monotherapy + ICI	5 (9.26%)	9 (16.07%)	
Dose reduction mode, *n* (%)			‐
Treatment cycles reduction	7 (12.96%)	Not applicable	
Dose reduction	44 (81.48%)	Not applicable	
Both	3 (5.56%)	Not applicable	
Treatment cycle, cycles	4 (2–6)	4 (2–27)	0.211
PD‐L1 tumor proportion score, *n* (%)			0.536
<1%	6 (11.11%)	10 (17.86%)	
1%–49%	12 (22.22%)	10 (17.86%)	
≥50%	13 (24.07%)	4 (7.14%)	
Unknown	23 (42.59%)	32 (67.14%)	
Local treatment, *n* (%)			0.518
Yes	18 (33.33%)	22 (39.29%)	
No	36 (66.67%)	34 (60.71%)	

Abbreviations: ECOG, Eastern Cooperative Oncology Group; GP, gemcitabine plus cisplatin; ICI, immune‐checkpoint inhibitor; PD‐L1, programmed cell death ligand‐1; TC, paclitaxel or nab‐paclitaxel plus carboplatin; TP, paclitaxel or nab‐paclitaxel plus cisplatin.

* *p* < 0.05.

### 3.2 Efficacy

**FIGURE 2:**
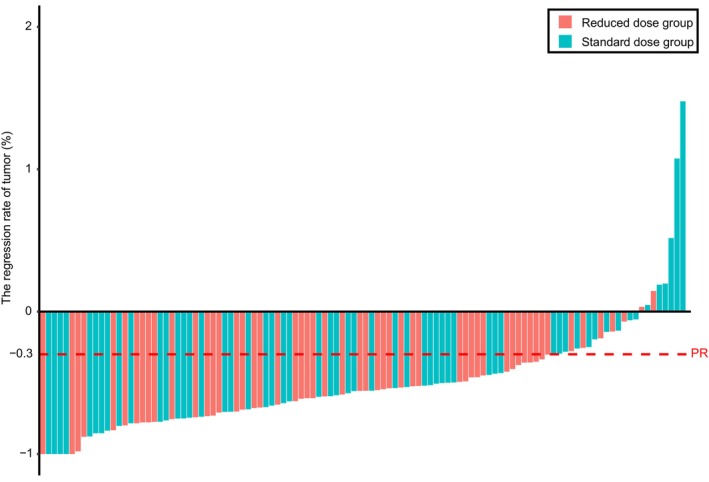
Assessment of response to reduced‐dose chemotherapy and standard‐dose chemotherapy combined with immunotherapy. The red dashed line indicates the threshold for a partial response (30% regression). PR, partial response.

As demonstrated in Table 2 and Figure 2, two patients (3.70%) in the reduced‐dose group and four patients (7.14%) in the standard‐dose group achieved CR during treatment. Among the patients with CR, two in the reduced‐dose group and one in the standard‐dose group achieved pathologic complete response (pCR). The ORR in the reduced‐dose group was similar to that in the standard‐dose group (85.19% vs. 71.43%, *p* = 0.082). Similar DCRs were observed in the reduced‐dose and standard‐dose groups (100% vs. 94.64%, *p* = 0.086). In the reduced‐dose group, no patients exhibited PD at the first efficacy evaluation resulting in a 100% DCR.

**TABLE 2:** Best overall response in the reduced‐dose group and standard‐dose group.

Characteristic	Reduced‐dose group	Standard‐dose group	*p*‐value
Best overall response, *n* (%)			
CR	2 (3.70%)	4 (7.14%)	0.429
PR	44 (81.48%)	36 (64.29%)	0.044*
SD	8 (14.81%)	13 (23.21%)	0.265
PD	0	3 (5.36%)	0.086
Objective response rate, %	85.19%	71.43%	0.082
Disease control rate, %	100%	94.64%	0.086

Abbreviations: CR, complete response; PD, progressive disease; PR, partial response; SD, stable disease.

* *p* < 0.05.

In most subgroup analyses of ORR and DCR, the reduced‐dose group exhibited similar ORR and DCR as the standard group (Figure [Link], [Link], and Table S1). However, in the subgroup analysis of ECOG score ≥1, both ORR and DCR were higher in the reduced‐dose group (ORR: 85.71% vs. 55.71, *p* = 0.005; DCR: 100% vs. 89.66%, *p* = 0.034). For stage IV, patients underwent subsequent‐line treatment and did not receive local treatment; the ORR was higher in the reduced‐dose group.

### 3.3 Safety

**FIGURE 3:**
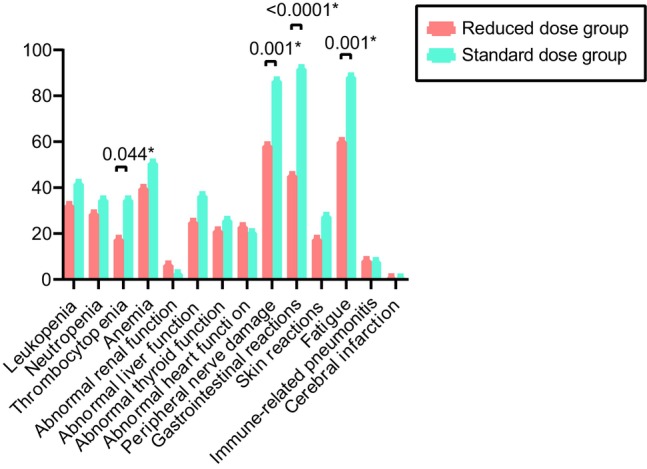
Graphic representation of the distribution of toxicities divided by chemotherapy dose groups and type of adverse events.

Regarding safety, most toxicities were grades 1–2 in both groups. As shown in Figure 3, the major toxic reactions were better alleviated in the reduced‐dose group than in the standard‐dose group, especially in the thrombocytopenia (*p* = 0.044), peripheral nerve damage (*p* = 0.001), gastrointestinal reactions (*p* < 0.0001), and fatigue (*p* = 0.001). Furthermore, we reported a lower incidence of grade 3–4 toxicity in the reduced‐dose group than in the standard‐dose group (27.78% vs. 42.86%, *p* = 0.100). Among patients in the reduced‐dose group, grade 3–4 neutropenia (eight cases, 14.81%), leukopenia (three cases, 5.5%), and anemia (three cases, 5.5%) were common. By contrast, in the standard group, the most common grade 3–4 toxicity in the standard group were anemia (11 cases, 19.64%), neutropenia (six cases, 10.7%), and leukopenia (six cases, 10.7%) (Table 3). We also report the incidence of cerebral infarction in this study. Notably, grade 3 cerebral infarction was observed in the standard group (one case, 1.7%). No toxicity‐related deaths occurred in the reduced‐dose group. However, the incidence of treatment‐emergent adverse events leading to death was 2.70% (one case) in the standard‐dose group. The patient died of a severe infection owing to grade 4 myelosuppression. Overall, reduced‐dose chemotherapy plus a PD‐1/PD‐L1 inhibitor was well tolerated by patients with LUSC.

**TABLE 3:** Incidence of treatment‐related adverse events.

Type of adverse events	Reduced‐dose group			Standard‐dose group		
	All grades *n* (%)	Grade 3 *n* (%)	Grade 4 *n* (%)	All grades *n* (%)	Grade 3 *n* (%)	Grade 4 *n* (%)
Leukopenia	18 (33.33%)	3 (5.5%)	0	24 (42.86%)	5 (8.9%)	1 (1.7%)
Neutropenia	16 (29.63%)	7 (12.9%)	1 (1.8%)	20 (35.71%)	6 (10.7%)	0
Thrombocytopenia	10 (18.52%)	1 (1.8%)	0	20 (35.71%)	3 (5.3%)	1 (1.7%)
Anemia	22 (40.74%)	3 (5.5%)	0	29 (51.79%)	10 (17.8%)	1 (1.7%)
Abnormal renal function	4 (7.4%)	0	0	2 (3.5%)	0	0
Abnormal liver function	14 (25.93%)	2 (3.7%)	1 (1.8%)	21 (37.5%)	2 (3.5%)	0
Abnormal thyroid function	12 (22.22%)	0	0	15 (26.79%)	1 (1.7%)	0
Abnormal heart function	13 (24.07%)	0	0	12 (21.43%)	1 (1.7%)	0
Peripheral nerve damage	32 (59.26%)	0	0	49 (87.5%)	0	0
Gastrointestinal reactions	25 (46.30%)	0	0	52 (92.86%)	3 (5.3%)	0
Skin reactions	10 (18.52%)	0	0	16 (28.57%)	1 (1.7%)	0
Fatigue	33 (61.11%)	0	0	50 (89.29%)	1 (1.7%)	0
Immune‐related pneumonitis	5 (9.26%)	2 (3.7%)	0	5 (8.9%)	1 (1.7%)	0
Cerebral infarction	1 (1.8%)	0	0	1 (1.7%)	1 (1.7%)	0

### 3.4 Survival analysis

**FIGURE 4:**
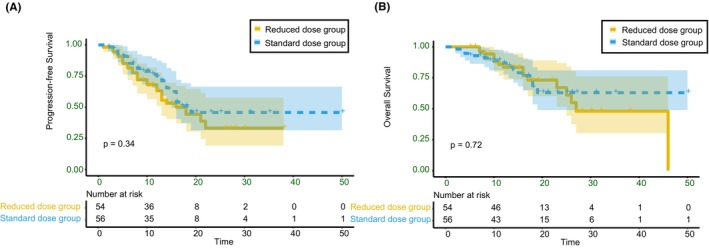
Progression‐free survival (A) and overall survival (B) in the reduced‐dose group and standard‐dose group, respectively.

**FIGURE 5:**
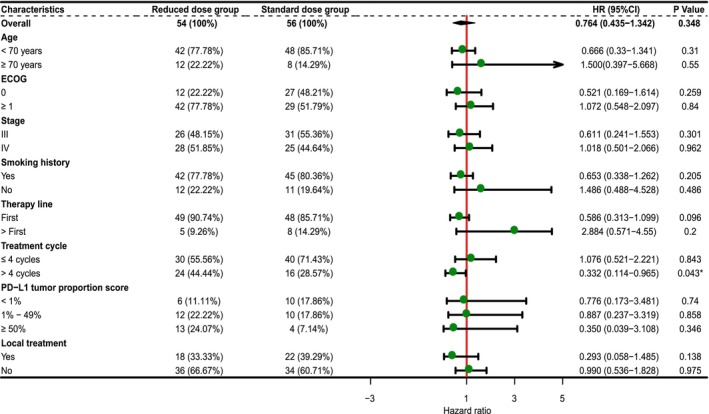
Subgroup analysis of progression‐free survival. ECOG, Eastern Cooperative Oncology Group; PD‐L1, programmed cell death ligand‐1.

**FIGURE 6:**
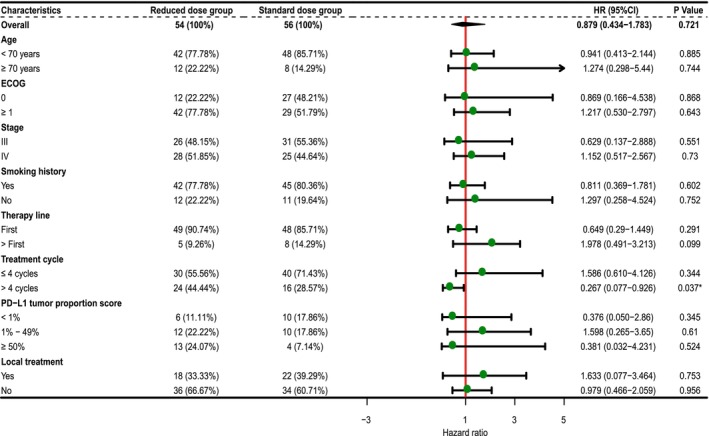
Subgroup analysis of overall survival. ECOG, Eastern Cooperative Oncology Group; PD‐L1, programmed cell death ligand‐1.

The median follow‐up period was 16 months (range, 4–50 months), and the final follow‐up was performed in February 2023. The median PFS was 12 months (range 1–38) in the reduced‐dose group and 12 months (range 2–50) in the standard‐dose group. In the reduced‐dose group, 11 patients (20.37%) developed intrapulmonary metastasis and recurrence, five (9.26%) developed mediastinal lymph node metastasis and recurrence, and 11 (20.37%) developed distant metastasis (liver, bone, distant lymph node, brain, and pancreas). In the standard group, 12 patients (21.43%) had intrapulmonary metastasis and recurrence, one (1.79%) had mediastinal lymph node metastasis, and nine (16.07%) had distant metastases (liver, bone, distant lymph node, brain, and pancreas). The median OS was 15 (range: 4–46) months in the reduced‐dose group and 16 (range 2–50) months in the standard‐dose group. The 12‐month PFS and OS rates were 63.3% and 83.5%, respectively, in the reduced‐dose group and 73.9% and 86.6%, respectively, in the standard group. The 24‐month PFS and OS rates were 33.3% and 67.1%, respectively, in the reduced‐dose group and 45.8% and 63%, respectively, in the standard group. There were no significant differences between the reduced‐dose group and standard‐dose group on PFS (*p* = 0.34) or OS (*p* = 0.72) (Figure 4). In the subgroup analyses of PFS and OS, the reduced‐dose group exhibited a PFS benefit similar to that of the standard group. We analyzed the differences in PFS and OS between the two subgroups for stage III and IV patients, patients receiving local and non‐local treatment, patients receiving first‐line treatment and subsequent‐line treatment, and patients receiving different regimens. The results were consistent with those previously mentioned (Figures 5 and 6, and Table S2).

## 4 DISCUSSION

In this study, we aimed to assess the efficacy and outcomes of combining reduced‐dose chemotherapy with immunotherapy in patients with LUSC. The combination of reduced‐dose chemotherapy and immunotherapy was effective and well tolerated. Furthermore, the modified regimen demonstrated similar efficacy to the full‐dose regimen used in previous RCTs for LUSC.

The results of the main randomized controlled trials (RCTs) on chemotherapy plus PD‐1/PD‐L1 inhibitors for LUSC are summarized in Table S3. The ORR, DCR, and PFS of the reduced‐dose group in our study were similar to those of a series of large‐scale phase III standard‐dose RCTs.^10^, ^11^, ^12^, ^13^, ^18^ Previous trials examining the clinical activity of PD‐1 inhibitors combined with full‐dose chemotherapy in patients with advanced LUSC reported ORR rates between 44.7% and 75% and median PFS between 5.1 months and 8.5 months. For instance, the Chinese RCT, RATIONALE‐307^12^, which examined tislelizumab plus carboplatin and paclitaxel (arm A) or nab‐paclitaxel (arm B), reported conflicting results with ORRs of 73% and 75% in arms A and B, respectively, and a median PFS of 7.6 months in both arms. In our study, the ORR was 85.19%, and the median PFS was 12 months in the reduced‐dose group. These findings indicate that the efficacy of the modified reduced‐dose chemotherapy plus immunotherapy regimen in the real world is similar to that of the full‐dose regimen in these RCTs. Interestingly, the reduced‐dose group had more patients with advanced age and high ECOG scores than the standard‐dose group. These results suggest that reduced‐dose chemotherapy, plus immunotherapy benefits elderly and frail patients. Similarly, the promising efficacy of reduced‐dose nab‐paclitaxel plus tislelizumab in elderly patients was corroborated in a phase II Chinese clinical trial.^17^ The study included patients with LUAD and LUSC; the ORR and PFS were 34.5% and 9.5 months (95% CI, 5.8–13.2), respectively. The ORR and PFS in our study were better than those reported in a phase II study. This finding suggests that reduced‐dose chemotherapy combined with PD‐1/PD‐L1 inhibitors can produce synergistic effects in patients with NSCLC. Furthermore, compared with single‐agent chemotherapy, the IPSOS study showed that first‐line treatment with atezolizumab provided a survival benefit and reduced toxicity in NSCLC patients ineligible for platinum‐based chemotherapy without EGFR or ALK alterations.^24^ The median OS in the reduced‐dose group in our study was similar to that in the atezolizumab monotherapy group in the IPSOS study (15 vs. 10.3 months). However, the IPSOS study reported a lower incidence of grade 3–4 toxicity compared with our study (16% vs. 27.78%). Because our study involved a combination regimen of chemoimmunotherapy, the higher grade 3–4 toxicity can be explained. The aforementioned studies demonstrated that adjusting treatment protocols and dosages to accommodate elderly and frail individuals is of great importance in the clinical practice of lung cancer. However, this finding warrants further investigation.

However, the mechanism underlying this potential synergistic effect has not been fully elucidated. Some studies have reported that cytotoxic agents can remodel the tumor immune microenvironment by inhibiting tumor‐induced immune suppression, inducing immunogenic cell death, and directing the stimulation of T‐cell responses.^25^, ^26^ Gaudreau et al. analyzed multi‐omics molecular and immune profiling data and found that chemotherapy can promote antitumor immunity through T‐ and B‐cell recruitment in the tumor immune microenvironment.^27^ In addition, a team reported higher densities of CD3+ lymphocytes and CD68+ tumor‐associated macrophages after chemotherapy in the tumor immune microenvironment.^28^ Regarding the potential mechanism by which reduced‐dose chemotherapy combined with immunotherapy can produce better efficacy, we speculate that, in the model of immunotherapy combined with reduced‐dose chemotherapy, reduced‐dose chemotherapy is crucial in affecting the tumor immune microenvironment, enhancing the efficacy of immunotherapy. This synergistic gain compensates for the diminished killing of tumor cells owing to the reduced dose of chemotherapy. The immunoregulatory effects of reduced‐dose chemotherapy are of particular interest.^21^, ^22^, ^29^, ^30^, ^31^, ^32^, ^33^, ^34^ After a stepwise investigation of LUSC cell lines, syngeneic murine models, and patient samples, He et al. observed that reduced‐dose chemotherapy contributed to the remodeling of an active immune microenvironment through the recruitment and activation of CD8+ T cells and DCs and the polarization of type I macrophages. They also found that the effects of routine chemotherapy appeared to be more additive than synergistic when combined with PD‐1/PD‐L1 inhibitors.^22^ Similarly, Pfirschke et al. demonstrated that reduced‐dose oxaliplatin combined with cyclophosphamide triggered immunogenic responses and improved immune activation.^25^ Nie et al. showed that a reduced dose of decitabine could promote T‐cell activation and strengthen the effectiveness and duration of clinical responses to anti‐PD‐1 antibodies in refractory classic Hodgkin lymphoma.^35^ Furthermore, reduced‐dose chemotherapy selectively increases the number of CD8+ T cells and enhances the antitumor immune response.^36^, ^37^ The role of reduced‐dose chemotherapy in regulating the tumor immune microenvironment may explain why the modified regimen produced good synergistic effects. However, because of the differences between human and mouse models, large trials should be conducted to assess further reduced‐dose combination therapy in patients with LUSC with poor physical status.

The AE profile observed in this study was as expected, based on the known AEs associated with PD‐1/PD‐L1 inhibitors (pembrolizumab, tislelizumab, sintilimab, camrelizumab, nivolumab, and durvalumab) and chemotherapeutic drugs (paclitaxel, nab‐paclitaxel, carboplatin, cisplatin, gemcitabine, and docetaxel). Furthermore, we report a novel safety signal event for cerebral infarction. Notably, grade 3 cerebral infarction was observed in the standard group (one case, 1.79%). Patients with malignancies are often hypercoagulable. Importantly, high‐dose chemotherapy for cancer is a significant risk factor for serious thromboembolic events.^38^ In addition, major toxic reactions were better alleviated in the reduced‐dose group than in the standard‐dose group. In RCTs examining standard‐dose chemotherapy plus immunotherapy, AEs grade ≥3 were reported in over 80% of patients, which was higher than the incidence of grade 3–4 AEs (27.78%) in our reduced‐dose group.^10^, ^11^, ^12^, ^13^, ^18^ All AEs were manageable.

We also performed subgroup analyses of the ORR and DCR. Our study indicates that frail patients can obtain good outcomes with reduced‐dose chemotherapy plus immunotherapy (population with ECOG score ≥1, stage IV, subsequent‐line treatment, and non‐local treatment). The reduced‐dose treatment regimen demonstrates less toxicity, leading to better patient adherence. By contrast, the best overall response in the three patients was PD in the standard‐dose group. Among them, two patients received second‐line treatment, and one received third‐line treatment. This finding suggests that some patients in the standard treatment group had more malignant tumors, explaining the poorer ORR in the standard treatment group in the subgroup analysis. Additionally, because of the small sample size of each subgroup, a more accurate analysis would require including a larger number of patients.

In addition, a post hoc interaction analysis of KEYNOTE‐407 indicated that the standard‐dose chemotherapy plus immunotherapy group had the same mPFS, regardless of PD‐L1 expression.^8^ Similarly, in this study, the mPFS of patients with PD‐L1 < 1%, PD‐L1 1%–49%, and PD‐L1 ≥ 50% were 11 (range 3–16) months, 11.5 (range 3–38) months, and 13 (range 4–21) months, respectively, in the reduced‐dose group. Therefore, we changed the scoring method for PD‐L1 expression.^39^ Patients also had the same mPFS when the scoring method for PD‐L1 expression was changed (PD‐L1 < 1% vs. PD‐L1 ≥ 1%, and PD‐L1 < 50% vs. PD‐L1 ≥ 50%). Both KEYNOTE‐407 and the results of our study suggest that immunotherapy is closely related to the molecular pathological characteristics of tumors. Patients with LUSC often have a high tumor mutational burden and strong immunogenicity; therefore, the immunotherapy response in patients with LUSC is less dependent on PD‐L1 expression.^40^ However, further studies are needed to establish a definite connection between the response to reduced‐dose chemotherapy plus immunotherapy and PD‐L1 expression levels.

Our study had some limitations, with the most prominent being that this was a single‐center study with a limited sample size. Despite this limitation, our study demonstrated promising efficacy and safety of reduced‐dose chemotherapy plus immunotherapy in patients with LUSC, which warrants further investigation. To enhance the robustness of our findings, we will continue enrolling more patients to expand the sample size. Additionally, it is notable that the PD‐L1 expression data were available for only a minority of patients, which is a critical factor in evaluating efficacy outcomes when immunotherapy is a therapy component. To address this issue in the future, we will save as many tissue and blood samples as possible from patients for additional efficacy marker testing. Furthermore, the observation duration was relatively short‐ and long‐term survival was not analyzed. Therefore, prospective large‐scale clinical trials are warranted.

## 5 CONCLUSIONS

In conclusion, our study demonstrates that a combination of reduced‐dose chemotherapy and immunotherapy exhibits promising efficacy in patients with LUSC. The efficacy of this modified regimen is similar to that of the full‐dose regimen in patients with LUSC. Notably, no unacceptable safety signs were identified with this modified regimen. Reduced‐dose chemotherapy combined with immunotherapy proves to be a treatment modality with fewer toxicities and better tolerability in patients with advanced and locally advanced LUSC. Therefore, further clinical trials exploring this approach would be worthwhile.

## AUTHOR CONTRIBUTIONS

**Ganlu Ouyang:** Data curation (equal); visualization (equal); writing – original draft (equal). **Yanyang Liu:** Conceptualization (equal); software (equal); writing – review and editing (equal). **Jiewei Liu:** Conceptualization (equal); writing – review and editing (equal). **Lin Huang:** Methodology (equal); software (equal). **Feng Luo:** Project administration (equal); supervision (equal). **Lu Li:** Project administration (equal); supervision (equal); writing – review and editing (equal).

## FUNDING INFORMATION

Project supported by the National Natural Science Foundation of China (82003089).

## CONFLICT OF INTEREST STATEMENT

The authors have no conflict of interest.

## ETHICS STATEMENT

Approval of the research protocol by an Institutional Reviewer Board: This study is approved by the Ethics Committee on Biomedical Research, West China Hospital of Sichuan University (2022–1742). Informed Consent: We confirm that informed consent was obtained from all participants and/or their legal guardians. Registry and the Registration No. of the study/trial: N/A. Animal Studies: N/A.

## Supporting information


Figure S1.

Figure S2.

Figure S3.

Figure S4.

Figure S5.

Figure S6.

Figure S7.

Figure S8.

Table S1.

Table S2.

Table S3.

## Data Availability

The datasets used and/or analyzed during the current study are available from the corresponding author on reasonable request.
