# Supplementary figures and images for: Efficacy and safety of reduced‐dose chemotherapy plus immunotherapy in patients with lung squamous cell carcinoma: A real‐world observational study
Source: Cancer Med. 2023 Sep 7;12(18):18679–90. doi: 10.1002/cam4.6478 (PMC10557858; doi:10.1002/cam4.6478)

## SUPPLEMENT FIGURE 1

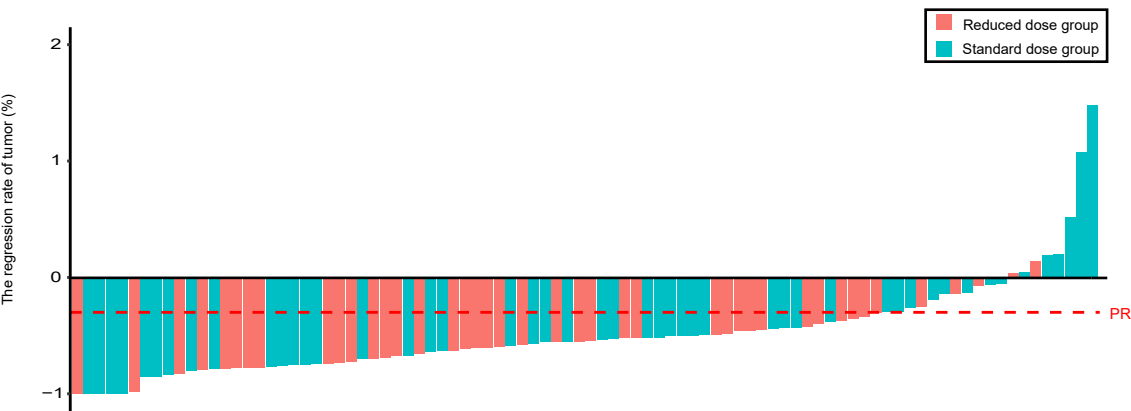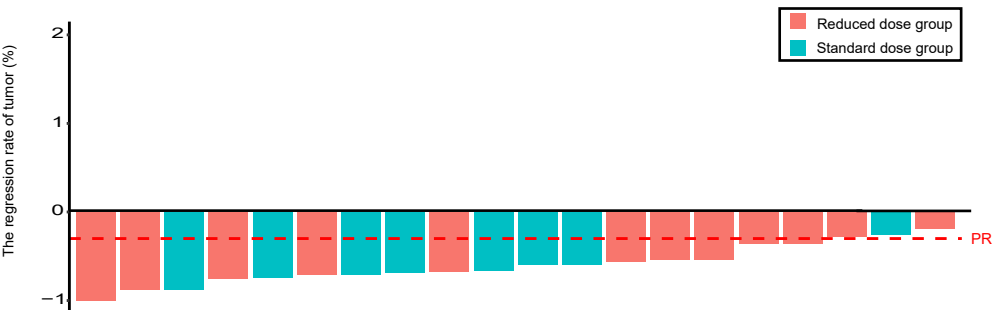

Supplement: Supplementary file 1 — Figure S1. [file CAM4-12-18679-s001.pdf]

**(A)**

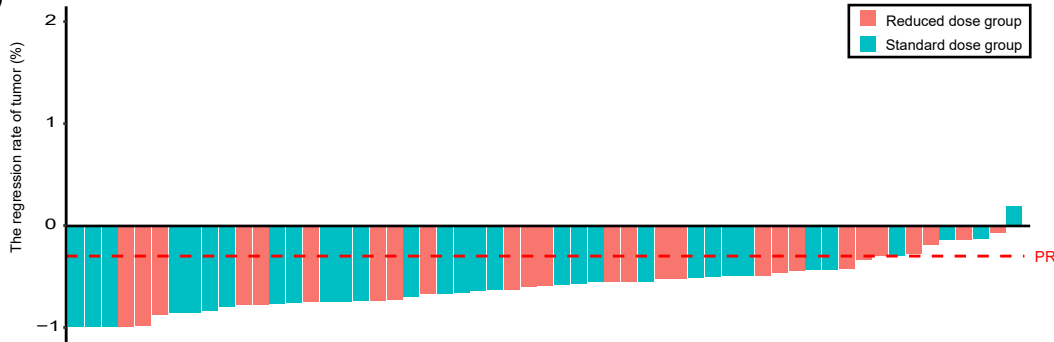

**(B)**

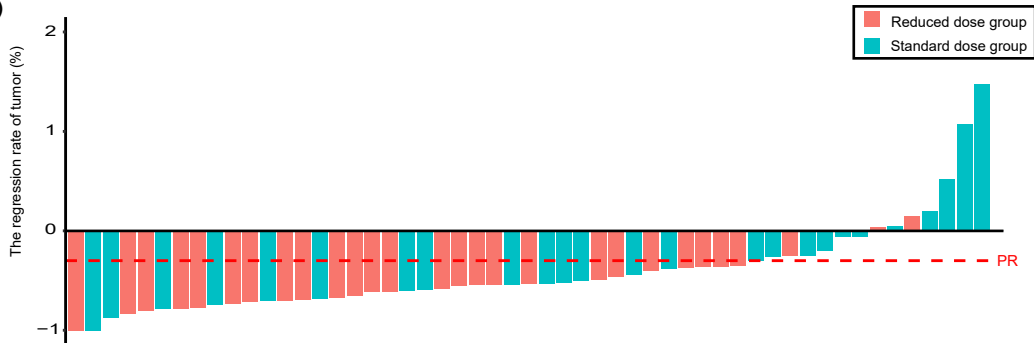

Supplement: Supplementary file 3 — Figure S3. [file CAM4-12-18679-s006.pdf]

(A)

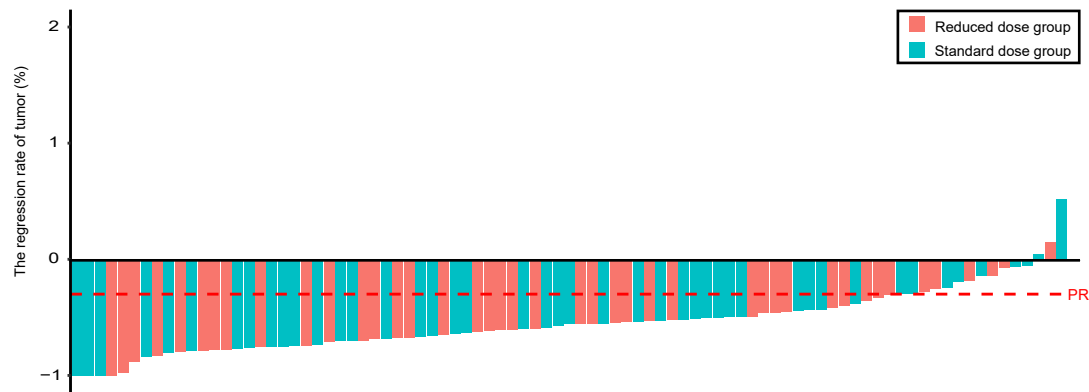

(B)

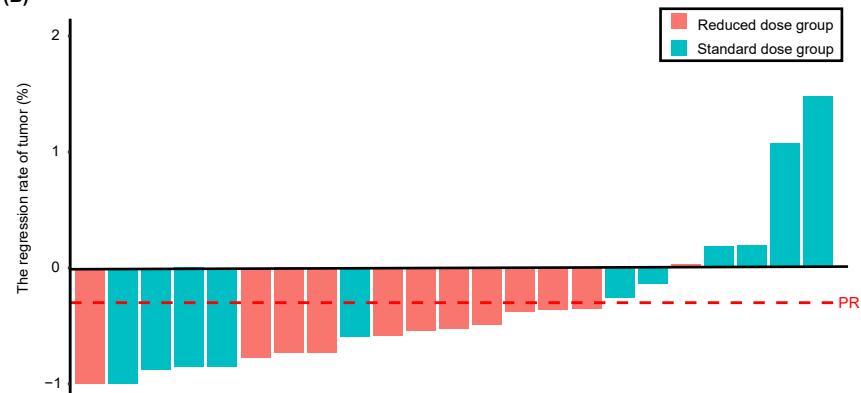

Supplement: Supplementary file 4 — Figure S4. [file CAM4-12-18679-s011.pdf]

SUPPLEMENT FIGURE 5

(A)

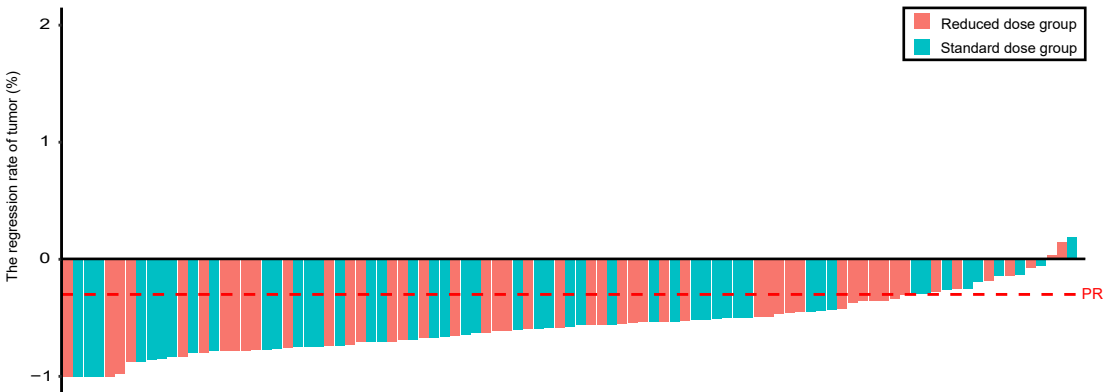

(B)

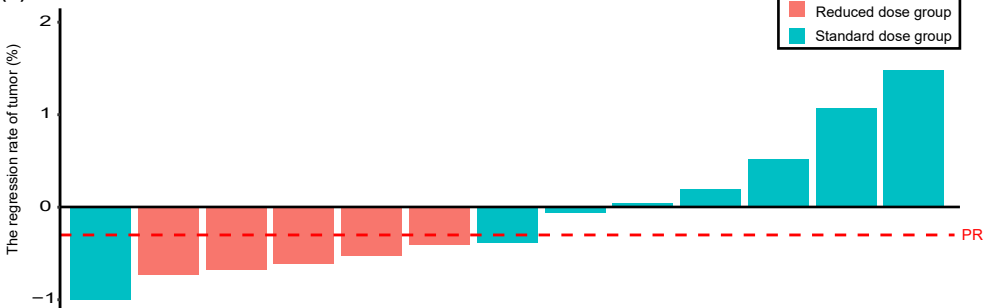

Supplement: Supplementary file 5 — Figure S5. [file CAM4-12-18679-s003.pdf]

SUPPLEMENT FIGURE 6

(A)

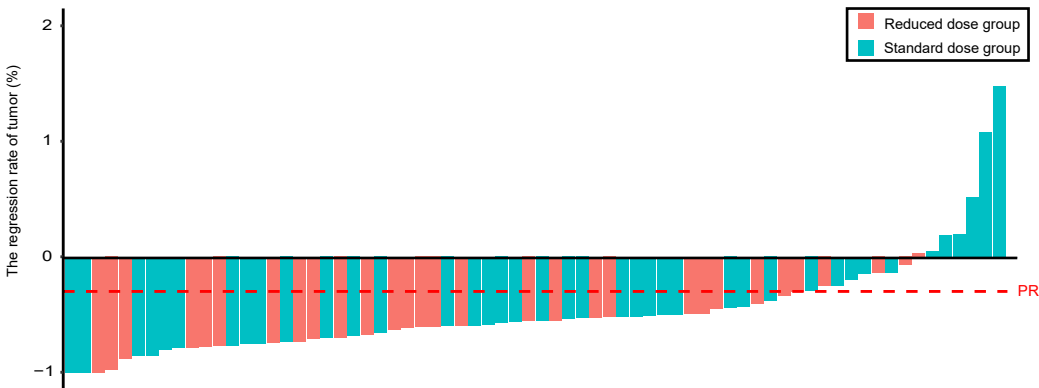

(B)

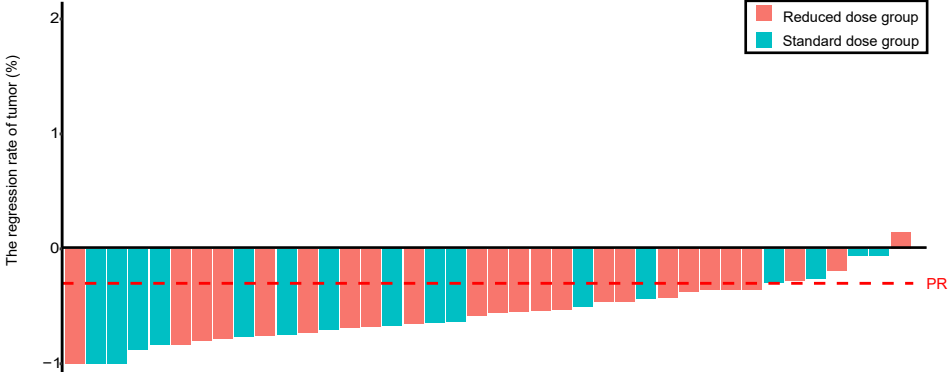

Supplement: Supplementary file 6 — Figure S6. [file CAM4-12-18679-s004.pdf]

(A)

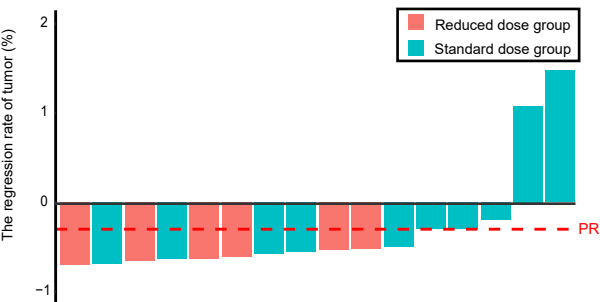

(B)

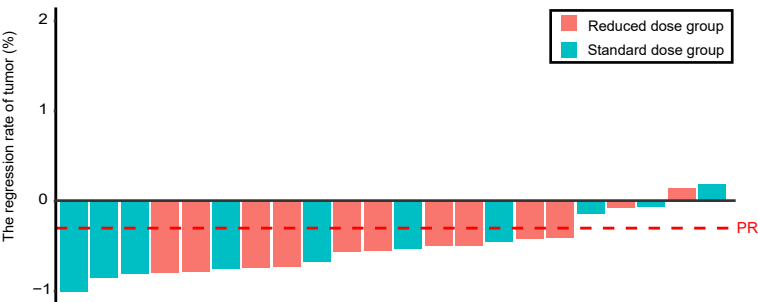

(C)

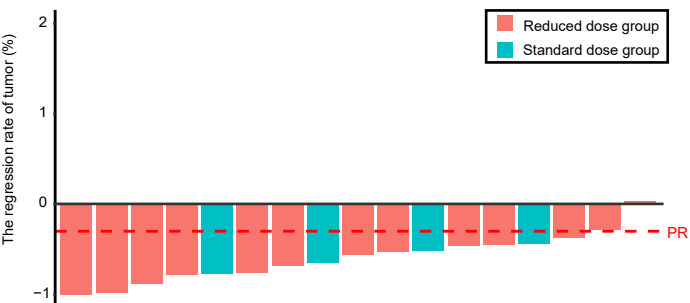

Supplement: Supplementary file 7 — Figure S7. [file CAM4-12-18679-s005.pdf]

SUPPLEMENT FIGURE 8

(A)

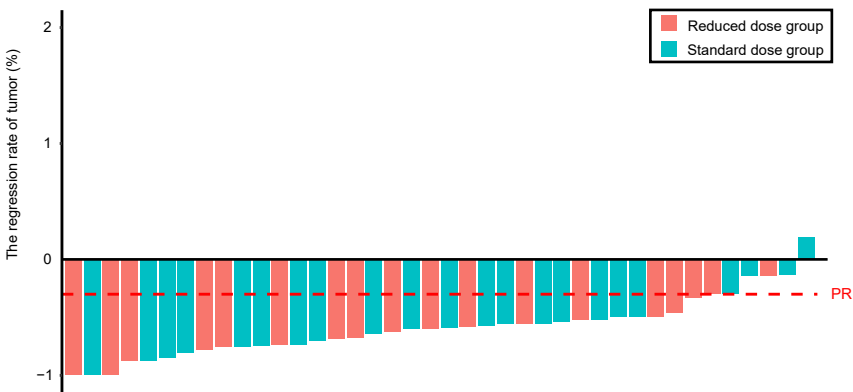

(B)

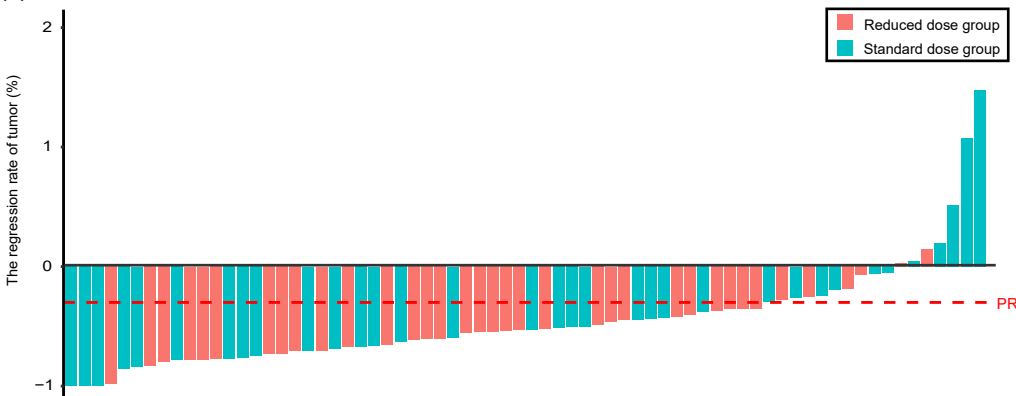

Supplement: Supplementary file 8 — Figure S8. [file CAM4-12-18679-s007.pdf]
