# Supplementary material for: Efficacy and safety of reduced‐dose chemotherapy plus immunotherapy in patients with lung squamous cell carcinoma: A real‐world observational study
Source: Cancer Med. 2023 Sep 7;12(18):18679–90. doi: 10.1002/cam4.6478 (PMC10557858; doi:10.1002/cam4.6478)
Supplement: Supplementary file 9 — Table S1. [file CAM4-12-18679-s010.docx]

Supplement Table1. Best overall response of subgroups.

| Subgroup | Objective response rate, % | | *P* value | Disease control rate, % | | *P* value |
| --- | --- | --- | --- | --- | --- | --- |
|  | Standard dose group | Reduced dose group |  | Standard dose group | Reduced dose group |  |
| Age |  |  |  |  |  |  |
| < 70 years | 68.75% | 85.71% | 0.059 | 93.75% | 100% | 0.101 |
| ≥ 70 years | 87.5% | 83.33% | 0.910 | 100% | 100% | 1.000 |
| ECOG |  |  |  |  |  |  |
| 0 | 88.89% | 83.33% | 0.799 | 100% | 100% | 1.000 |
| ≥ 1 | 55.17% | 85.71% | 0.005* | 89.66% | 100% | 0.034* |
| Stage |  |  |  |  |  |  |
| III | 90.32% | 80.77% | 0.305 | 100% | 100% | 1.000 |
| IV | 48% | 89.29% | 0.001* | 88% | 100% | 0.062 |
| Smoking history |  |  |  |  |  |  |
| Yes | 75.56% | 83.33% | 0.374 | 97.78% | 100% | 0.334 |
| No | 54.55% | 73.91% | 0.134 | 81.82% | 100% | 0.203 |
| Therapy line |  |  |  |  |  |  |
| First | 81.25% | 83.67% | 0.605 | 100% | 100% | 1.000 |
| ≥ First | 12.5% | 100% | 0.006* | 62.5% | 100% | 0.284 |
| Treatment cycle |  |  |  |  |  |  |
| ≤ 4 cycles | 70% | 83.33% | 0.201 | 92.5% | 100% | 0.128 |
| > 4 cycles | 75% | 87.5% | 0.521 | 100% | 100% | 1.000 |
| PD-L1 tumor proportion score |  |  |  |  |  |  |
| < 1% | 60% | 100% | 0.220 | 80% | 100% | 0.562 |
| 1% - 49% | 60% | 83.33% | 0.381 | 100% | 100% | 1.000 |
| ≥ 50% | 100% | 84.62% | 0.703 | 100% | 100% | 1.000 |
| Local treatment |  |  |  |  |  |  |
| Yes | 90.91% | 88.89% | 0.925 | 100% | 100% | 1.000 |
| No | 58.82% | 83.33% | 0.024* | 91.18% | 100% | 0.071 |

ECOG, Eastern Cooperative Oncology Group; PD-L1, programmed cell death ligand-1.

* *P*<0.05.
