# Supplementary material for: Efficacy and safety of reduced‐dose chemotherapy plus immunotherapy in patients with lung squamous cell carcinoma: A real‐world observational study
Source: Cancer Med. 2023 Sep 7;12(18):18679–90. doi: 10.1002/cam4.6478 (PMC10557858; doi:10.1002/cam4.6478)
Supplement: Supplementary file 10 — Table S2. [file CAM4-12-18679-s009.docx]

Supplement Table2. The survival analysis of subgroups.

| Subgroup | mPFS (months) | | *P* value | mOS (months) | | *P* value |
| --- | --- | --- | --- | --- | --- | --- |
|  | Standard dose group | Reduced dose group |  | Standard dose group | Reduced dose group |  |
| Age |  |  |  |  |  |  |
| < 70 years | 13.5 (2-50) | 12.5 (1-38) | 0.31 | 17 (2-50) | 15 (5-46) | 0.885 |
| ≥ 70 years | 8 (4-15) | 10 (4-18) | 0.55 | 9 (7-26) | 13.5 (8-23) | 0.744 |
| ECOG |  |  |  |  |  |  |
| 0 | 13 (4-50) | 10 (1-27) | 0.259 | 16 (5-50) | 12.5 (9-27) | 0.868 |
| ≥ 1 | 10 (2-31) | 12.5 (3-38) | 0.84 | 16 (2-35) | 16 (5-46) | 0.643 |
| Stage |  |  |  |  |  |  |
| III | 14 (2-35) | 14 (3-30) | 0.301 | 17 (2-35) | 15 (7-32) | 0.551 |
| IV | 8 (2-50) | 10 (1-38) | 0.962 | 13 (3-50) | 12 (5-46) | 0.73 |
| Smoking history |  |  |  |  |  |  |
| Yes | 14 (2-50) | 12 (3-30) | 0.205 | 17 (2-50) | 15.5 (5-46) | 0.602 |
| No | 7 (2-31) | 13 (1-38) | 0.486 | 10 (4-31) | 14.5 (8-38) | 0.752 |
| Therapy line |  |  |  |  |  |  |
| First | 13 (2-35) | 11 (1-38) | 0.096 | 17 (2-35) | 15 (5-46) | 0.291 |
| ≥ First | 5 (2-50) | 13 (6-26) | 0.2 | 14 (3-50) | 11 (6-26) | 0.099 |
| Treatment cycle |  |  |  |  |  |  |
| ≤ 4 cycles | 10.5 (2-31) | 12 (1-38) | 0.843 | 13 (2-35) | 17 (7-38) | 0.344 |
| > 4 cycles | 16.5 (6-50) | 10 (3-18) | 0.043* | 18 (7-50) | 12.5 (5-46) | 0.037* |
| PD-L1 tumor proportion score |  |  |  |  |  |  |
| < 1% | 14.5 (2-19) | 11 (3-16) | 0.74 | 17.5 (4-35) | 15 (10-16) | 0.345 |
| 1% - 49% | 13.5 (5-50) | 11.5 (3-38) | 0.858 | 14 (5-50) | 11.5 (7-38) | 0.61 |
| ≥ 50% | 18.5 (12-31) | 13 (4-21) | 0.346 | 23.5 (12-31) | 15 (6-32) | 0.524 |
| Local treatment |  |  |  |  |  |  |
| Yes | 13 (5-25) | 14 (5-30) | 0.138 | 14.5 (5-25) | 14 (5-30) | 0.753 |
| No | 11.5 (2-50) | 10 (1-38) | 0.975 | 16 (2-50) | 15 (7-46) | 0.956 |

ECOG, Eastern Cooperative Oncology Group; PD-L1, programmed cell death ligand-1.

* *P*<0.05.
