# Supplementary material for: Efficacy and safety of reduced‐dose chemotherapy plus immunotherapy in patients with lung squamous cell carcinoma: A real‐world observational study
Source: Cancer Med. 2023 Sep 7;12(18):18679–90. doi: 10.1002/cam4.6478 (PMC10557858; doi:10.1002/cam4.6478)
Supplement: Supplementary file 11 — Table S3. [file CAM4-12-18679-s008.docx]

Supplement Table3. Summary of the main randomized controlled trials on chemotherapy plus PD-1/PD-L1 inhibitor for LUSC.

| Clinical trial | Study design | ORR | DCR | mPFS (months) |
| --- | --- | --- | --- | --- |
| KEYNOTE-407 | Carboplatin+paclitaxel or nab-paclitaxel±Pembrolizumab | 62.6% | 86% | 8 (6.3-8.4) |
| ORIENT-12 | Platinum+gemcitabine±Sintilimab | 44.7% | - | 5.1 (4.9-5.7) |
| CameL-SQ | Carboplatin+paclitaxel±Camrelizumab | 64.8% | - | 8.5 (6.9-10.4) |
| RATIONALE-307 | Carboplatin+paclitaxel or nab-paclitaxel±Tislelizumab | 73%, 75% | 88%, 91% | 7.6 (6.0-9.8) ,  7.6 (5.8-11.0) |
| IMpower-131 | Carboplatin+nab-paclitaxel±Atezolizumab | 49.7% | 81% | 6.3 (5.7-7.1) |

PD-1, programmed cell death protein-1; PD-L1, programmed cell death ligand-1; LUSC, lung squamous cell carcinoma; ORR, objective response rate; DCR, disease control rate; PFS, progression-free survival.
