# Supplementary material for: Efficacy and safety of reduced‐dose chemotherapy plus immunotherapy in patients with lung squamous cell carcinoma: A real‐world observational study
Source: Cancer Med. 2023 Sep 7;12(18):18679–90. doi: 10.1002/cam4.6478 (PMC10557858; doi:10.1002/cam4.6478)

SUPPLEMENT FIGURE 2

(A)

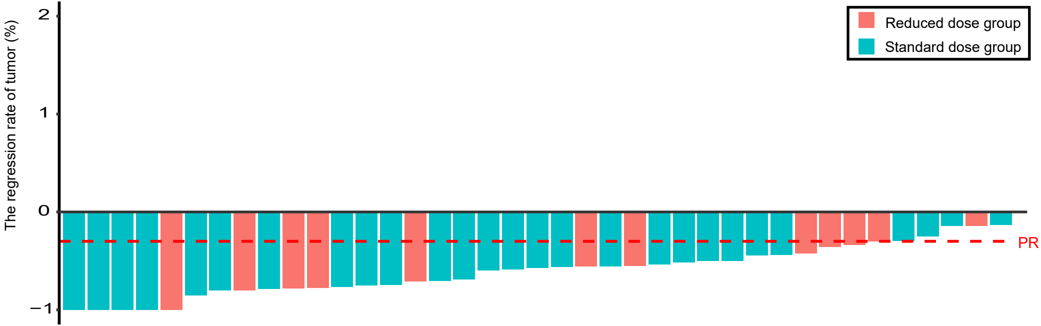

(B)

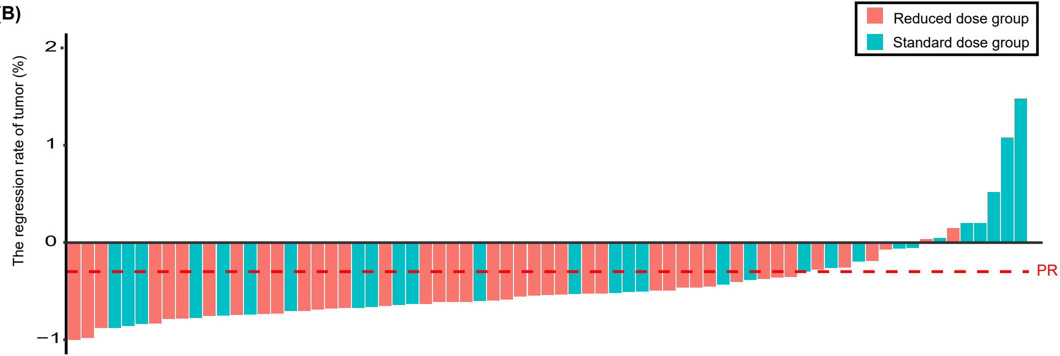

Supplement: Supplementary file 2 — Figure S2. [file CAM4-12-18679-s002.pdf]
